# Proteomic analysis of acquired tamoxifen resistance in MCF-7 cells reveals expression signatures associated with enhanced migration

**DOI:** 10.1186/bcr3144

**Published:** 2012-03-14

**Authors:** Changhua Zhou, Qiu Zhong, Lyndsay V Rhodes, Ian Townley, Melyssa R Bratton, Qiang Zhang, Elizabeth C Martin, Steven Elliott, Bridgette M Collins-Burow, Matthew E Burow, Guangdi Wang

**Affiliations:** 1 Department of Chemistry, Xavier University of Louisiana, 1 Drexel Drive, New Orleans, LA 70125, USA; 2 Blood Research Laboratory, Chengdu Blood Center, Chengdu, Sichuan 610041, China; 3 RCMI Cancer Research Program, Xavier University of Louisiana, 1 Drexel Drive, New Orleans, LA 70125, USA; 4 Department of Medicine, Tulane University School of Medicine, 1415 Tulane Ave., New Orleans, LA 70112, USA; 5 Department of Pharmacology, Tulane University School of Medicine, 1415 Tulane Ave., New Orleans, LA 70112, USA

## Abstract

**Introduction:**

Acquired tamoxifen resistance involves complex signaling events that are not yet fully understood. Successful therapeutic intervention to delay the onset of hormone resistance depends critically on mechanistic elucidation of viable molecular targets associated with hormone resistance. This study was undertaken to investigate the global proteomic alterations in a tamoxifen resistant MCF-7 breast cancer cell line obtained by long term treatment of the wild type MCF-7 cell line with 4-hydroxytamoxifen (4-OH Tam).

**Methods:**

We cultured MCF-7 cells with 4-OH Tam over a period of 12 months to obtain the resistant cell line. A gel-free, quantitative proteomic method was used to identify and quantify the proteome of the resistant cell line. Nano-flow high-performance liquid chromatography coupled to high resolution Fourier transform mass spectrometry was used to analyze fractionated peptide mixtures that were isobarically labeled from the resistant and control cell lysates. Real time quantitative PCR and Western blots were used to verify selected proteomic changes. Lentiviral vector transduction was used to generate MCF-7 cells stably expressing S100P. Online pathway analysis was performed to assess proteomic signatures in tamoxifen resistance. Survival analysis was done to evaluate clinical relevance of altered proteomic expressions.

**Results:**

Quantitative proteomic analysis revealed a wide breadth of signaling events during transition to acquired tamoxifen resistance. A total of 629 proteins were found significantly changed with 364 up-regulated and 265 down-regulated. Collectively, these changes demonstrated the suppressed state of estrogen receptor (ER) and ER-regulated genes, activated survival signaling and increased migratory capacity of the resistant cell line. The protein S100P was found to play a critical role in conferring tamoxifen resistance and enhanced cell motility.

**Conclusions:**

Our data demonstrate that the adaptive changes in the proteome of tamoxifen resistant breast cancer cells are characterized by down-regulated ER signaling, activation of alternative survival pathways, and enhanced cell motility through regulation of the actin cytoskeleton dynamics. Evidence also emerged that S100P mediates acquired tamoxifen resistance and migration capacity.

## Introduction

Acquired resistance to hormone therapy remains a major challenge in the treatment of estrogen receptor positive (ER(+)) metastatic breast cancers. Previous studies have demonstrated that ER (+) breast cancer can escape anti-estrogen actions by up-regulating other signaling pathways involved in cell survival and proliferation. Enhanced signaling via growth factor receptors, such as EGFR [1] and HER2 [2], has been implicated in acquired resistance to endocrine therapy. Activation of downstream intracellular signaling like the MAPK pathway and the PI3K/Akt pathway has also been linked to hormone resistance [3,4]. The cross-talk between ER and such alternative signaling pathways are believed to enable breast cancer to evade the antiproliferative effects of anti-estrogens [5]. This knowledge has led to numerous treatment strategies combining endocrine and targeted inhibitor therapies. However, early clinical trials of EGFR- and ERBB2-targeted inhibitors (for example, gefitinib, erlotinib, trastuzamab, and lapatinib) or m-TOR inhibitors (everolimus and temsirolimus) in combination with endocrine therapies have yielded mixed results [6-8]. It is likely that cross-talk and negative feedback loops may result in cellular resistance to individual inhibitors [9]. Additional therapies targeting converging points of shared signaling pathways, such as MYC and cyclin D1-CKD4, may be more effective at blocking proliferation in resistant breast cancers [10].

Current understanding of endocrine resistance mechanisms is largely based on the study of relatively few genes. Integrative approaches that examine gene expression in the genomic and proteomic context may lead to the discovery of previously unconsidered mechanisms for the modulation of therapeutic responses. The current study employed a quantitative proteomic strategy to capture global changes in protein expression in a tamoxifen resistant cell line derived from the wild type MCF-7 parental cells. *In vitro *studies of tamoxifen resistance have provided valuable foundational data that can be translated into *in vivo *and clinical applications [11-13]. The most widely used and best characterized cell line for study of acquired tamoxifen resistance has been the MCF-7 variants, from which much of our current understanding of the mechanisms of hormone resistance has derived [13,14]. While numerous earlier studies in other laboratories have demonstrated that tamoxifen resistant breast cancer cell lines were generated by long term exposure of MCF-7 cells to 10^-6 ^to 10^-7^M 4-OH Tam over a period of 6 to 12 months, adaptive signatures of the resulting resistant phenotypes may vary with different experimental conditions employed. For example, EGFR expression was reported to be 10-fold higher in one tamoxifen-resistant model [14] but not in other models [15,16]. It has also been shown [13] that use of dextran coated charcoal-stripped (DCC) serum in tamoxifen treatment may introduce, in addition to adaptive changes of the cells in response to tamoxifen, effects of long term estrogen deprivation (LTED), thus complicating the interpretation of molecular signals of resistance development for tamoxifen. Moreover, in estrogen deprived medium, tamoxifen can act as an agonist [17] towards ER, adding another complicating factor to the mechanistic interpretation of tamoxifen resistance. We used a phenol-red free DMEM medium containing 5% FBS so that the background estrogen level is in a range that is unlikely to induce adaptive changes due to estrogen deprivation and to minimize the agonistic action of tamoxifen in ER(+) breast cancer cells.

In this study, we examined global proteomic alterations of the tamoxifen resistant cell line vs the parental MCF-7 cells using an isobaric labeling approach combined with a high resolution tandem mass spectrometry instrument for relative quantitative analysis. Our proteomics data demonstrated extensive adaptive changes in the proteome involving hundreds of significantly up- and down-regulated proteins. In particular, results from this study revealed the overexpression of multiple tumorigenic, pro-metastatic proteins and the down-regulation of ER mediated signaling pathways. These findings provide novel insights into the complex events of the adaptive signaling network occurring during the acquisition of tamoxifen resistance in breast cancer cells and highlight the role of S100P in conferring both resistance and enhanced migration.

## Materials and methods

### Cell culture

MCF-7 cell line was purchased from ATCC (ATCC #HTB-22, Manassas, VA, USA), and routinely cultured in phenol red-free DMEM medium supplemented with 5% FBS, 4 mM glutamine, 1 mM sodium pyruvate, 100 IU/mL penicillin, 100 μg/mL streptomycin and 0.25 μg/mL amphotericin. Tamoxifen resistant variant cells (MCF-7-TamR) derived from MCF-7 cells were continuously cultured in the medium as described above containing additional 10^-7 ^M 4-OH Tam (Sigma-Aldrich, St Louis, MO, USA) for at least six months, along with the parental MCF-7-cells under identical culture conditions except that the control cells were treated with 0.1% ethanol. The two cell lines were grown side by side at all times. Cultures were maintained in 5% carbon dioxide at a temperature of 37°C.

### Cell growth assay

For growth assay in the presence of 10^-7 ^M 4-OH Tam, MCF-7 cells cultured with 10^-7 ^M 4-OH Tam for zero to six months were plated in six-well plates at a density of 50,000 in each well in 5% FBS DMEM medium. The cells were then treated with 10^-7 ^M 4-OH Tam for five days, while equal treatment volumes of ethanol were used as a vehicle control. Cell numbers were counted with a Coulter instrument (Beckman-Coulter, Indianapolis, IN, USA). The ratio of 4-OH Tam treated cell numbers to vehicle treated cell numbers was defined as survival ratio. Experiments were conducted in triplicate and data represented as mean ± SD.

For dose-dependent proliferation assays, MCF-7-TamR and MCF-7-control cells were seeded in 96-well plate with a density 3,000 per well and treated with varying concentrations (10^-7 ^to 10^-5 ^M) of 4-OH tamoxifen for five days; 0.1% ethanol was used as a vehicle control. Alamar Blue dye (Invitrogen, Grand Island, NY, USA) was added and incubated for 2 h at 37°C, protected from light. A Synergy 2 microplate reader (BioTek, Winooski, VT, USA) was used to record fluorescence using an excitation wavelength at 560 nm and emission wavelength at 590 nm. The ratio of 4-OH Tam-treated cell fluorescence intensity to that of vehicle treated cells was determined as the survival ratios in triplicate experiments. Data were represented as mean ± SD.

## Colony formation assay

Colony formation assays were conducted as outlined previously [18-20]. MCF-7-control or MCF-7-TamR cells were cultured in 5% FBS phenol red-free DMEM. Cells were then plated at a density of 2,000 cells per well in 2 ml 5% FBS DMEM in six-well plates (Falcon, Franklin Lakes, NJ, USA) and allowed to adhere overnight at 37°C, 5% CO_2_. The next day cells were treated with 4-OH Tam (100 nM). Equal treatment volumes of dimethyl sulfoxide (DMSO) were used as a vehicle control. Cells were allowed to grow until control treatment colonies reached > 50 cells per colony (approximately 10 to 14 days). Colonies were then fixed with glutaraldehyde for 30 minutes, stained with crystal violet (0.1% in 20% methanol) for 30 minutes and washed. Colony number was determined manually. Experiments were conducted in triplicate and data represented as mean ± SEM.

## Cell lysis

MCF-7-TamR and MCF-7-control cells were cultured to 80% confluent in the medium as described above, and washed with cold Hank's Buffered Salt Solution (HBSS) for three times, then collected with a cell scraper. NP40 cell lysis buffer (Invitrogen) containing additional 1 mM of phenylmethylsulfonyl fluoride (PMSF) and protease inhibitor cocktail (Sigma) was used to extract total cellular proteins. The concentration of proteins was measured with BCA assay (Pierce Biotechnology, Rockford, IL, USA). The cell lysis was stored at -80°C before further processing.

### Trypsin digestion

Protein samples were digested with sequencing grade modified trypsin (Promega Corp. Madison, WI, USA) according to the manufacturer's instructions. Briefly, to aliquots of 100 μg of protein sample was added 45 μL of 200 mM triethyl ammonium bicarbonate (TEAB) and the final volume was adjusted to 100 μL with ultrapure water. A total of 5 μL of 200 mM tris(2-carboxyethyl)phosphine (TCEP) was added and the resulting mixture was incubated for 1 h, then 5 μL of 375 mM iodoacetamide was added and the mixture was incubated for 30 minutes without light. After incubation, 1 mL of pre-chilled acetone was added and the precipitation was allowed to proceed overnight. The acetone-precipitated protein pellets were suspended with 100 μL of 200 mM TEAB and 2.5 μg of trypsin was added to digest the sample overnight at 37°C.

### Tandem Mass Tags (TMT) labeling

Tandem mass tags TMT^6 ^(Thermo Scientific, Rockford, IL, USA) with different molecular weights (126 to approximately 131 Da) were applied as isobaric tags for relative and absolute quantification. According to the manufacturer's protocols, the digested samples were individually labeled with TMT^6 ^reagents for 1 h as follows: three 100-μg aliquots of digested MCF-7-control peptides were each labeled with a different isobaric tag (TMT126, 127 and 128, respectively). Likewise, 100-μg aliquots of peptides from MCF-7-TamR cells were labeled with TMT129, 130, and 131 mass tags, respectively. The labeling reaction was quenched with 5% hydroxylamine. Finally, the six labeled peptide aliquots were combined for subsequent fractionation.

### Fractionation of labeled peptide mixture using a strong cation exchange column

The combined TMT labeled peptide mixture was fractionated with a strong cation exchange column (SCX) (Thermo Scientific) on a Shimadzu 2010 HPLC equipped with a UV detector (Shimadzu, Columbus, MD, USA). Mobile phase consists of buffer A (5 mM KH_2_PO_4_, 25% acetonitrile, pH 2.8) and buffer B (buffer A plus 350 mM KCl). The column was equilibrated with Buffer A for 30 minutes before sample injection. The mobile phase gradient was set as follows at a flow rate of 1.0 mL/minute: (a) 0 to 10 minutes: 0% buffer B; (b) 10 to 40 minutes: 0% to 25% Buffer B, (c) 40 to 45 minutes: 25% to 100% Buffer B; (d) 45 to 50 minutes: 100% buffer B; (e) 50 to 60 minutes: 100% to 0% buffer B; (f) 60 minutes to 90 minutes: 0% buffer B. A total of 60 fractions were initially collected, lyophilized and combined into 15 final fractions based on SCX chromatographic peaks.

### Desalination of fractionated samples

A C_18 _solid-phase extraction (SPE) column (Hyper-Sep SPE Columns, Thermo-Fisher Scientific, Waltham, MA, USA) was used to desalt all collected fractions. The combined 15 fractions were each adjusted to 1-mL final volume containing 0.25% (v/v in water) trifluoroacetic acid (TFA, Sigma). The C_18 _SPE columns were conditioned before use by filling them with 1 mL acetonitrile and allowing the solvent to pass through the column slowly (approximately three minutes). The columns were then rinsed three times with 1 mL 0.25% (v/v in water) TFA solution. The fractions were loaded on to the top of the SPE cartridge and allowed to elute slowly. Columns were washed four times with 1-mL 0.25% TFA aliquots before the peptides were eluted with 3 × 400 μL of 80% acetonitrile/0.1% formic acid (aqueous).

### LC-MS/MS analysis on LTQ-Orbitrap

Peptides were analyzed on an LTQ-Orbitrap XL instrument (Thermo-Fisher Scientific) coupled to an Ultimate 3000 Dionex nanoflow LC system (Dionex, Sunnyvale, CA, USA). High mass resolution was used for peptide identification and high energy collision dissociation (HCD) was employed for reporter ion quantification. The RP-LC system consisted of a peptide Cap-Trap cartridge (0.5 × 2 mm) (Michrom BioResources, Auburn, CA, USA) and a pre-packed BioBasic C_18 _PicoFrit analytical column (75 μm i.d. × 15 cm length, New Objective, Woburn, MA, USA) fitted with a FortisTip emitter tip. Samples were loaded onto the trap cartridge and washed with mobile phase A (98% H_2_O, 2% acetonitrile and 0.1% formic acid) for concentration and desalting. Subsequently, peptides were eluted over 180 minutes from the analytical column via the trap cartridge using a linear gradient of 6 to 100% mobile phase B (20% H_2_O, 80% acetonitrile and 0.1% formic acid) at a flow-rate of 0.3 μL/minute using the following gradient: 6% B for 5 minutes; 6 to 60% B for 125 minutes; 60 to 100% B for 5 minutes; hold at 100% B for 5 minutes;100 to 6% B in 2 minutes; hold at 6% B for 38 minutes.

The LTQ-Orbitrap tandem mass spectrometer was operated in a data-dependent mode. Briefly, each full MS scan (60,000 resolving power) was followed by six MS/MS scans where the three most abundant molecular ions were dynamically selected and fragmented by collision-induced dissociation (CID) using a normalized collision energy of 35%, and the same three molecular ions were also scanned three times by HCD-MS^2 ^with collision energy of 45%. MS scans were acquired in profile mode and MS/MS scans in centroid mode. LTQ-Orbitrap settings were as follows: spray voltage 2.0 kV, 1 microscan for MS1 scans at 60, 000 resolution (fwhm at *m*/*z *400), microscans for MS^2 ^at 7,500 resolution (fwhm at *m*/*z *400); full MS mass range, *m*/*z *400 to 1,400; MS/MS mass range, *m*/*z *100 to 2,000. The "FT master scan preview mode", "Charge state screening", "Monoisotopic precursor selection", and "Charge state rejection" were enabled so that only the 2+, 3+ and 4+ ions were selected and fragmented by CID and HCD.

### Database search and TMT quantification

The protein search algorithm used was Mascot (Matrix Science, Boston, MA, USA). Mascot format files were generated by the Proteome Discoverer 1.2 software (Thermo-Fisher Scientific) using the following criteria: database, *IPI_Human.fasta.v3.77*; enzyme, trypsin; maximum missed cleavages, 2; Static modifications, carbamidomethylation (+57 Da), N-terminal TMT6plex (+229 Da), lysyl TMT6plex (+229 Da). Dynamic modifications, N-terminal Cln- pyro-Glu(+17Da); methionine oxidation (+16 Da); STY phosphorylation (+80 Da); MS peptide tolerance was set at 15 ppm; MS/MS tolerance at 0.05 Da. Peptides reported by the search engine were accepted only if they met the false discovery rate of *P *< 0.05 (target decoy database). For TMT quantification, the ratios of TMT reporter ion abundances in MS/MS spectra generated by HCD (up to six reporter ions ranging from *m*/*z *126.12 to m/z 131.14) from raw data sets were used to calculate fold changes in proteins between control and treatment.

### Quantitative RT-PCR

#### Confirmation of selected targets identified in proteomic analysis

Total RNA from MCF-7-TamR and control cells was extracted using a PureLink total RNA purification system (Invitrogen) and quantitatively analyzed with a nanodrop spectrophotometer (Thermo Scientific). The reverse transcription was carried out with a SuperScript first-strand synthesis system (Invitrogen) using Oligo(dT)_12-18 _primers. The primer pairs used to amplify the genes were designed using the online tool of Oligo Perfect Designer (Invitrogen), and beta actin (actb) was employed as an internal standard. Primer specificity was confirmed by BLAST analysis. For real-time PCR analyses, a MyiQ real time PCR detection system (BioRad, Hercules, CA, USA) and a SYBR GreenER qPCR supermix kit (Invitrogen) were used as follows: 50°C for 2 minutes, 95°C for 8 minutes and 30 seconds, and 50 cycles (15 seconds at 95°C, 1 minute at 60°C). The data were analyzed with a normalized gene expression method (ΔΔCt) using the iQ5 Optical System Software (BioRad), and the gene actb was used as a reference for normalization. All experiments were repeated three times independently.

#### ER regulated gene transcripts

MCF-7-control or MCF-7-TamR cells were seeded at a density of 2 × 10^6 ^cells per 25 cm^2 ^culture flask in phenol red-free 5% FBS-DMEM. On the following day, cells were washed in PBS and media were changed to phenol red-free media supplemented with 5% CS-DMEM and grown to 50 to 80% confluency for 48 h before treatment with vehicle (DMSO), 17β-estradiol (100 pM), or tamoxifen (100 nM). RNA was extracted using QiaShredders (QIAGEN, Valencia, CA, USA) and purified on RNeasy columns (QIAGEN) according to the manufacturer's protocol. RNA quality and concentration were determined by absorbance at 260 and 280 nm. Then 2 μg of total RNA was reverse transcribed using the iScript kit (Bio-Rad Laboratories). The levels of ERα, PgR and SDF-1 transcripts were determined using real-time quantitative PCR. The primer sequences are as follows (sense and antisense, respectively): PgR, 5'-TACCCGCCCTATCTCAACTACC-3', 5'-TGCTTCATCCCCACAG-ATTAAACA-3'; SDF-1, 5'-AGTCAGGTGGTGGCTTAACAG-3', 5'-AGAGGAGGTGAAGGCAGTGG-3'; and ER_*α*_, 5'-GGCATGGTGGAGATCTTCGA-3', 5'-CCTCTCCCTGCAGATTCATCA-3', Actin, 5'- TGA GCG CGG CTA CAG CTT -3', 5'-CCTTAATGTCACACACGATT-3'. The PCR reaction was carried out as follows: step 1: 95°C 3 minutes; step 2: for 40 cycles 95°C 20 seconds, 60°C 1 minute; step 3: 70°C 10 seconds, held at 4°C. Each reaction tube contained: 12.5 μL 2 × SYBR Green supermix + 6.5 μL nuclease-free water + 1 μL 0.1 μg/μL primer (pair) + 5 μL cDNA (0.2 μg/μL). Genes were amplified in triplicate. Data were analyzed by comparing relative target gene expression to actin control. Relative gene expression was analyzed using 2-ΔΔCt method [21].

### Western blot

MCF-7-control or MCF-7-TamR cells were seeded in 10 cm^2 ^plates at a density of 60 to 70% confluence (5 to 10 × 10^6 ^cells) and were allowed to grow for three days until they approached 80 to 90% confluence. The media was then removed and the cells were scraped into 1 mL of PBS plus 3 mM EDTA. The cell suspensions were spun for five minutes at 2,000 × g and the supernatant was aspirated. The cell pellets were lysed by vortexing in 200 μL of M-PER mammalian protein extraction buffer (Pierce, cat. # 78501) containing protease and phosphatase inhibitors (Sigma, cat. #'s P1860-1ML, P0044, and P5726). The samples were then spun in a microcentrifuge for five minutes at 12,000 × g and the supernatants were collected. Protein concentrations were determined using a nanodrop spectrophotometer (Thermo Life Sciences) and 50 μg of total protein was loaded and run on a 4 to 12% polyacrylamide gel (Invitrogen). The gels were blotted onto nitrocellulose using the iblot transfer system (Invitrogen). The blots were blocked for one hour at room temperature in 1 × TBST (Affymetrix, Santa Clara, CA, USA, cat # 77500 5 LT) containing 5% non-fat milk. The blots were then washed in 1 × TBST and were incubated overnight at 4°C in 10 mL of primary antibody at a 1:500 dilution in 5% BSA/TBST (Sigma cat # A7906-1 KG). Blots were then washed in 1 × TBST and incubated with infrared-labeled secondary antibodies (LiCor) for 30 minutes at room temperature. The blots were then washed in 1 × TBST and scanned using the Odyssey infrared imaging system (LiCor, Lincoln, NE USA). Bands were quantified using the Odyssey software (LiCor) and normalized to bands corresponding to the housekeeping Rho-GDI protein. Four independent samples were prepared for each cell line. Paired *t *test analyses were performed for each protein using Origin 8.5.1 software (control vs TamR), and *P-*values < 0.05 were considered significant.

### Transwell migration assay

Migration assays were performed following the manufacturer's instructions (BD Falcon, Sparks, MD, USA). Briefly, MCF-7-control or MCF-7-TamR cells were seeded at a density of 2.5 × 10^4 ^in 500 μL serum-free and phenol red-free media in the upper chamber of a 24-well transwell system. Phenol red-free DMEM supplemented with FBS (5%) was used as a chemoattractant in the lower wells. After 24 h, membranes were scrubbed, fixed with 10% phospho-buffered formalin, permeabilized with 100% ice-cold methanol, and stained with 0.1% crystal violet in 20% methanol. Membranes were removed and mounted on glass slides for visualization by light microscopy. Data are represented as a percent of the migrated MCF-TamR cells per 100 × field of view (100×) ± SEM for triplicate experiments.

### MCF-7 cells overexpressing S100P

#### Construction of S100P lentiviral vector

The S100P gene was generated by elongating RT-PCR using a Superscript III one-step RT-PCR system (Invitrogen) with the following primers: S100P-F (sense) 5'-CGC CAC CAT GAC GGA ACT AGA GAC AGC C-3' and S100P-R (antisense) 5'-GGA TCC TCA TTT GAG TCC TGC CTT CTC-3'. The RT-PCR reaction was carried out as follows: step 1: 45°C for 30 minutes and 94°C for 2 minutes; step 2: 35 cycles at 94°C for 15 sec, 51°C for 30 sec and 72°C for 1 minute; step 3: 72°C for 5 minutes and held at 4°C. The PCR product was cloned using a TA Cloning kit (Invitrogen). The S100P lentiviral vector (pLenti6/S100P) was constructed by digesting vector pLenti6 (Invitrogen) with *EcoR I and BamH I *for insertion of the S100P gene.

#### MCF-7-S100P cell line stably overexpressing S100P

To produce S100P-overexpressing lentivirus, the 293FT cells were co-transfected with expression construct (pLenti6/S100P) and the optimized packaging mix (ViraPower Packaging mix, Invitrogen) from a lentiviral expression system (Invitrogen). The transfection was carried out by incubating cells overnight at 37°C in a CO_2 _incubator using a Lipofectamine 2000 reagent (Invitrogen). Media were replaced in 24 hours and the virus-containing supernatants were harvested and centrifuged at 48 to 72 hours. MCF-7 cells were grown to 30 to 50% confluent, and the culture medium was replaced with viral supernatants as obtained previously. Polybrene was added for the overnight viral transfection. Subsequently, medium was replaced every 2 to 3 days with antibiotic (Blasticidin) and the selection process continued for a total of 10 to 12 days. The stable MCF-7-S100P cell line was cultured in phenol red free DMEM medium with 5% FBS, and the S100P expression was checked with Western blot.

### Bioinformatics and statistics

Bioinformatics were performed on significantly altered proteins. This was determined by two parameters: one is having an analytical replication *P*-value of < 0.05 and the second is determined by the ratio value. The standard deviation (SD) of all the ratios in the control sample was determined and then significance was defined as (1 +/- 2SD) [22-24]. Classification of proteins was determined by the web program PANTHER [25]. The proteins were analyzed for over expression of gene ontology terms in the categories of pathways, molecular function and biological process. Pathway mapping was done using Pathvisio 2.0.11, a tool for visualizing and editing biological pathways [26]. The ratio data of the significant proteins were loaded into Pathvisio and used to map onto preloaded pathways from Wikipathways [27] and KEGG [28-30]. The pathway thus created was heavily modified from KEGG pathway 04810, "Regulation of actin cytoskeleton" in *Homo sapiens*.

### Patient survival analysis

An online database [31] was used to assess relevance of significantly changed protein expressions to relapse-free survival. The database was established using gene expression data and survival information on 1,809 patients downloaded from Gene Expression Omnibus (GEO) (Affymetrix HGU133A and HGU133+2 microarrays, Santa Clara, CA, USA). Briefly, single or multiple genes were entered into the database to obtain Kaplan-Meier survival plot where the number-at-risk was indicated below the main plot. Hazard ratio (and 95% confidence intervals) and logrank *P *were calculated and displayed on the webpage. For the genes listed in Tables 1 and 2, their effects on relapse-free survival (RFS) were calculated and listed. Positive logrank *P*-values indicate positive correlation (that is, either overexpression or down-regulation of a gene correlates with decreased survival) and negative logrank *P*-values indicate negative correlation (that is, either up- or down-regulation of a gene is associated with increased survival).

**Table 1:** Selected up-regulated proteins in tamoxifen resistant breast cancer cells

Accession	# AAs	MW (kDa)	Description	Gene symbols	Fold change TamR/Ctrl	t-test (*P-*values)	Relapse free survival analysis (Logrank *P*)
IPI00025311	584	61.7	Breast carcinoma-amplified sequence 1	bcas1	11.28	1.3E-06	-3.2E-5
IPI00017526	95	10.4	Protein S100-P	**s100p**	5.20	2.3E-08	1.7E-6
IPI00218831	218	25.7	Glutathione S-transferase Mu 1	gstm1	3.70	8.6E-07	-3.8E-11
IPI00183695	97	11.2	Protein S100-A10	**s100a10**	3.15	1.0E-07	2.3E-5
IPI00922108	1002	111.1	Integrin alpha-V	itgav	2.88	7.8E-06	2.0E-9
IPI00021267	976	108.2	Ephrin type-A receptor 2	**epha2**	2.81	3.1E-05	-2.0E-9
IPI00027341	348	38.5	Macrophage-capping protein	capg	2.80	2.3E-07	3.9E-5
IPI00106687	222	25.7	Latexin	lxn	2.69	1.3E-05	0.2
IPI00013895	105	11.7	Protein S100-A11	**s100a11**	2.51	3.0E-05	5.1E-10
IPI00455315	339	38.6	Annexin A2	**anxa2**	2.41	2.7E-08	0.93
IPI00297910	323	35.7	Tumor-associated calcium signal transducer 2	**tacstd2**	2.19	1.1E-06	0.53
IPI00903145	583	68.5	Radixin	**rdx**	2.17	2.4E-06	0.25
IPI00219301	332	31.5	Myristoylated alanine-rich C-kinase substrate	**marcks**	2.14	6.9E-06	-0.17
IPI00853146	167	19.2	Caveolin	cav	2.08	1.0E-03	-4.2E-8
IPI00215995	1051	116.5	Integrin alpha-3	itga3	2.00	2.0E-04	-3.9E-9
IPI00010414	329	36.0	PDZ and LIM domain protein 1	pdlim1	1.94	1.8E-06	-0.009
IPI00795633	448	52.3	Clusterin	clu	1.88	1.5E-04	-0.02
IPI00010214	104	11.7	Protein S100-A14	s100a14	1.87	2.1E-04	0.96
IPI00465431	250	26.1	Galectin-3	**lgals3**	1.79	2.5E-06	0.28
IPI00219219	135	14.7	Galectin-1	**lgals1**	1.78	2.0E-04	0.008
IPI00018364	183	20.5	Ras-related protein Rap-2b	rap2b	1.71	7.5E-05	6.7E-6
IPI00016485	394	42.7	Protein phosphatase slingshot homolog 3	ssh3	1.68	1.1E-03	-2.4E-11
IPI00217563	798	88.4	Integrin beta-1	itgb1	1.64	3.8E-08	6.9E-12
IPI00180240	44	5.1	Thymosin beta-4-like protein 3	tmsl3	1.63	3.7E-07	1.7E-5
IPI00641181	195	19.5	MARCKS-related protein	marcksl1	1.55	3.6E-06	0.011
IPI00759759	327	37.1	Epidermal growth factor receptor kinase substrate 8-like protein 2	eps8l2	1.53	2.4E-03	-0.13
IPI00021828	98	11.1	Cystatin-B	**cstb**	1.52	4.2E-04	4.7E-5
IPI00001871	340	36.5	PRKC apoptosis WT1 regulator protein	pawr	1.47	1.9E-04	0.23
IPI00843975	586	69.4	Ezrin	**ezr**	1.41	4.0E-04	1.8E-5
IPI00847442	92	10.1	FK506 binding protein12	fkbp12	1.40	1.4E-05	0.0011
IPI00022462	760	84.8	Transferrin receptor protein 1	tfrc	1.40	1.2E-05	0.00049
IPI00005585	124	13.7	Tax1-binding protein 3	tax1bp3	1.39	5.5E-06	6.7E-6
IPI00016179	98	11.5	Protein S100-A13	**s100a13**	1.39	7.6E-09	0.15
IPI00005202	247	26.2	Membrane-associated progesterone receptor component 2	pgrmc2	1.38	2.2E-04	3.6E-5
IPI00015148	184	20.8	Ras-related protein Rap-1b	rap1b	1.36	5.7E-06	2.3E-5
IPI00291175	1066	116.6	Vinculin	vcl	1.35	2.3E-05	-0.11
IPI00375426	323	36.2	Cathepsin H	ctsh	1.34	3.6E-04	-0.44
IPI00011285	714	81.8	Calpain-1 catalytic subunit	capn1	1.33	6.1E-06	-0.22
IPI00020599	417	48.1	Calreticulin	calr	1.31	3.3E-06	0.67
IPI00009342	1657	189.1	Ras GTPase-activating-like protein IQGAP1	iqgap1	1.29	7.9E-07	1.0E-4
IPI00010080	527	58.0	Serine/threonine-protein kinase OSR1	oxsr1	1.28	5.0E-04	0.49
IPI00478231	193	21.8	Transforming protein RhoA	**rhoa**	1.28	4.8E-07	0.016
IPI00012011	166	18.5	Cofilin-1	**cfl1**	1.28	1.5E-05	-7.9E-8
IPI00025084	268	28.3	Calpain small subunit 1	capn2	1.26	1.4E-05	-1.6E-12
IPI00013808	911	104.8	Alpha-actinin-4	actn4	1.24	1.5E-04	0.64
IPI00000513	821	90.9	E-cadherin	cdh1	1.24	6.6E-05	1.5E-7
IPI00220739	195	21.7	Membrane-associated progesterone receptor component 1	pgrmc1	1.23	1.1E-03	5.7E-7
IPI00028091	418	47.3	Actin-related protein 3	actr3	1.23	4.8E-04	2.4E-14
IPI00217519	206	23.6	Ras-related protein Ral-A	rala	1.21	3.6E-03	< 1.0E-16
IPI00922213	1014	111.2	fibronectin 1	fn1	1.19	3.4E-02	0.46
IPI00220847	1745	194.3	Integrin beta-4	itgb4	1.19	6.3E-04	-0.12
IPI00016786	191	21.2	Cell division control protein 42 homolog	cdc42	1.15	5.2E-05	-4.3E-10

**Table 2:** Selected down-regulated proteins in tamoxifen resistant breast cancer cells

Accession	#AAs	MW [kDa]	Description	Gene symbols	Fold Change TamR/Ctrl	t-test (p values)	Relapse free survival analysis (Logrank P)
IPI00218414	260	29.2	Carbonic anhydrase 2	**ca2**	-2.70	1.3E-05	0.79
IPI00032808	219	24.3	Ras-related protein Rab-3D	rab3d	-2.36	4.5E-06	1.2E-4
IPI00472076	175	19.4	tumor protein D53	tpd52l1	-2.03	7.5E-06	0.66
IPI00012866	480	55.7	RAC-alpha serine/threonine-protein kinase	akt1	-1.95	3.4E-06	-0.028
IPI00647268	189	21.6	Ras homolog gene family, member C	rhoc	-1.86	6.4E-06	-0.019
IPI00025318	114	12.8	SH3 domain-binding glutamic acid-rich-like protein	sh3bgrl	-1.75	2.4E-05	3.8E-5
IPI00550020	102	11.5	Parathymosin	**ptms**	-1.74	5.2E-06	0.095
IPI00186008	291	33.0	PCTP-like protein	**stard10**	-1.74	9.7E-04	NA
IPI00011564	198	21.6	Syndecan-4	sdc4	-1.68	2.1E-04	NA
IPI00019502	1960	226.4	Myosin-9	myh9	-1.63	5.8E-06	-0.085
IPI00011696	845	98.3	Proto-oncogene vav	vav1	-1.58	4.8E-02	6.2E-11
IPI00022283	84	9.1	Trefoil factor 1	tff1	-1.54	1.5E-04	1.5E-5
IPI00550900	172	19.6	Translationally-controlled tumor protein	tpt1	-1.51	1.4E-06	1.1E-10
IPI00019345	184	21.0	Ras-related protein Rap-1A	rap1a	-1.50	1.2E-05	-9.5E-8
IPI00479997	149	17.3	Stathmin	stmn1	-1.47	1.5E-05	-2.9E-12
IPI00011229	412	44.5	Cathepsin D	**ctsd**	-1.45	5.9E-07	0.56
IPI00216319	246	28.2	14-3-3 protein eta	**ywhah**	-1.45	5.5E-04	-0.066
IPI00217975	586	66.4	Lamin-B1	lmnb1	-1.43	2.3E-05	0.32
IPI00414676	724	83.2	Heat shock protein HSP 90-beta	hsp90ab1	-1.40	5.9E-07	5.6E-7
IPI00246975	225	26.5	Glutathione S-transferase Mu 3	gstm3	-1.37	8.9E-07	0.008
IPI00009607	183	20.7	Ras-related protein Rap-2c	rap2c	-1.36	2.4E-03	-3.1E-6
IPI00020904	358	40.9	Serine/threonine-protein kinase PRKX	prkx	-1.33	6.5E-03	0.7
IPI00000041	196	22.1	Rho-related GTP-binding protein RhoB	rhob	-1.33	2.0E-03	0.0032
IPI00003815	204	23.2	Rho GDP-dissociation inhibitor 1	arhgdia	-1.27	1.5E-03	0.0042
IPI00955014	297	34.1	cell division control protein 2 homolog	cdk1	-1.26	6.1E-04	NA
IPI00419235	229	26.9	Glutathione S-transferase Mu 5	gstm5	-1.25	1.3E-04	< 1E-16
IPI00941907	350	38.4	Serine-threonine kinase receptor-associated protein	strap	-1.25	1.7E-05	-5.4E-9
IPI00297261	435	49.9	Tyrosine-protein phosphatase non-receptor type 1	ptpn1	-1.25	4.3E-04	1.1E-11
IPI00019812	499	56.8	Serine/threonine-protein phosphatase 5	ppp5c	-1.15	4.4E-04	7.4E-12

## Results

### Establishment of 4-hydroxytamoxifen resistant cell line, MCF-7-TamR

**Figure 1:**
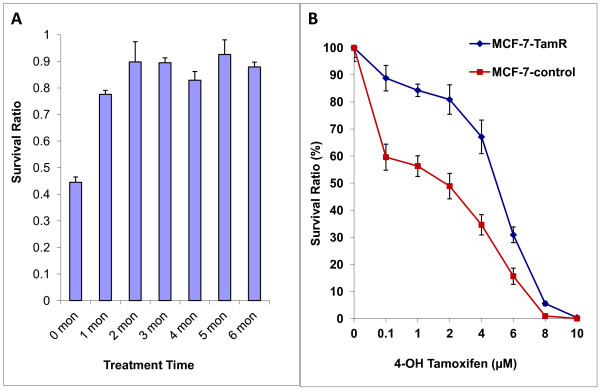
**Treatment of MCF-7 cells with 4-OH Tam resulted in a resistant phenotype**. **A**. Survival ratio vs time (zero to six months). MCF-7 cells cultured with 10^-7 ^M 4-OH tamoxifen for zero to six months were plated in six-well plates at a density of 50,000 per well in 5% FBS DMEM medium. The cells were counted after treatment with 10^-7 ^M 4-OH tamoxifen for five days; the final concentration of ethanol was 0.1%. **B**. Survival ratio vs 4-hydroxytamoxifen concentration. MCF-7 cells cultured for six months with 10^-7 ^M 4-OH tamoxifen (MCF-7-TamR) and 0.1% ethanol (MCF-7-control) were seeded in 96-well plates at a density of 3,000 each in 5% FBS phenol-red free DMEM medium. The cells were then treated with 10^-7 ^to 10^-5 ^M 4-OH tamoxifen for five days.

Cell growth assays were performed to determine the acquired resistance of MCF-7 cells in response to continuous exposure to 4-hydroxytamoxifen over a period of six months. Initially, MCF-7 cells showed greater than 50% growth inhibition with tamoxifen treatment as measured by survival ratio. As shown in Figure 1A, the survival ratio of the tamoxifen-treated MCF-7 cells was approximately 45%. By the end of the first month, the ratio reached 75%. The survival ratio increased further to 90% by the end of month 2, indicating that tamoxifen-treated cells have resumed the growth rate comparable to untreated conditions. The survival ratio of the tamoxifen treated cell line remained at about 90% from month 3 and beyond.

The acquired resistance to tamoxifen was further measured by dose-dependent growth assays (Figure 1B). When both MCF-7-control and MCF-7-TamR cells were treated with 4-OH Tam at increasing concentrations from 10^-7 ^M to 10^-5 ^M, the survival ratios showed marked differences between the two cell lines. For instance, at 100 nM 4-OH Tam, MCF-7-TamR cells maintained a 90% survival ratio, compared to 60% for the tamoxifen sensitive MCF-7-control cells. At 4 μM, the ratio dramatically decreased to 30% for the control cells but remained around 70% for MCF-7-TamR. This trend continued until 4-OH Tam concentration reached 10 μM where no cells survived from either cell line (Figure 1B).

**Figure 2:**
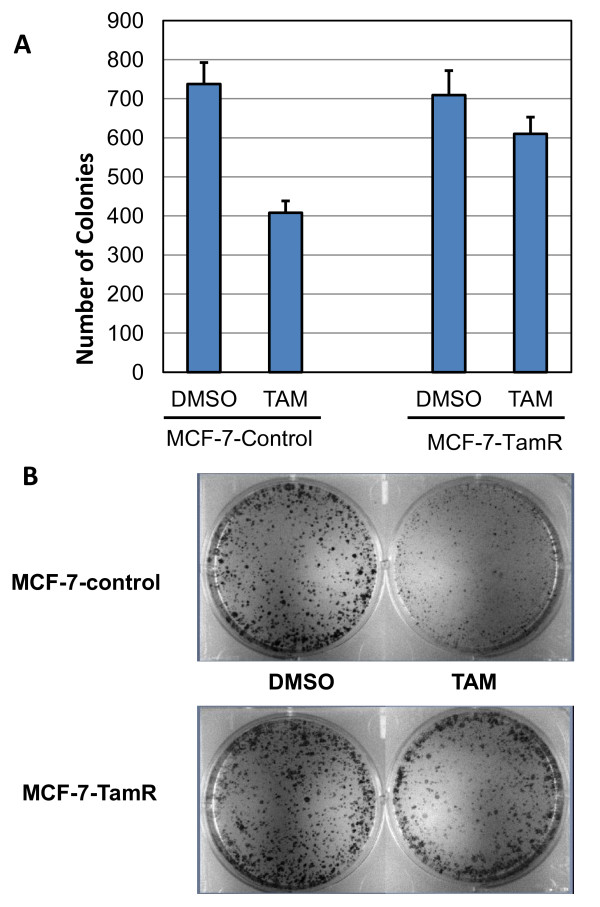
**MCF-7-TamR cells are resistant to tamoxifen in clonogenic assays**. **A**. Effect of tamoxifen on colony formation of MCF-7-TamR and MCF-7-control. Number of colonies formed in resistant and sensitive cell lines was plotted as a function of treatment (DMSO and tamoxifen). **B**. Representative colony images of the resistant and sensitive cells.

To further investigate the proliferative behavior of the resistant cell line clonogenic assays were also performed. MCF-7-control and MCF-7-TamR cells were each treated with vehicle (DMSO) or 100 nM 4-OH Tam. The proliferation of tamoxifen sensitive MCF-7 cells was significantly inhibited in the presence of 100 nM 4-OH Tam (approximately 400 colonies compared to 720 in DMSO, Figure 2A). In contrast, the MCF-7-TamR cells demonstrated strong resistance to 4-OH Tam induced inhibition of colony formation. Shown in Figure 2B is a representative image of colony formation of the two cell lines treated with vehicle (DMSO) and 4-OH Tam, respectively.

### Proteomics data reveal extensive changes in expression associated with acquired tamoxifen resistance

To increase the total number of proteins that can be identified and quantified in whole cell lysates, we used a gel-free approach that relies on isobaric mass tag labeling for quantitative analysis and a combination of two-dimensional HPLC separation and high resolution mass spectrometry for maximal peptide detection and identification. Indeed, this approach yielded a total of 2,128 identified and 2,088 quantified proteins, which represent five to six times more proteins than were analyzed by a 2D-gel based approach used in our previous study [32]. Of these proteins, over 1,200 were found to have statistically significant changes (*P *< 0.05) in expression in the tamoxifen resistant cell line (Additional file 1 Table S1). While this number appears high, it reflects the high confidence in the analytical reproducibility because the *P-*values were calculated from the three isobaric labels as analytical replicates for each cell line sample. Thus, some of the smaller fold changes in protein expression, while statistically significant and accurately reflective of the relative protein quantities in the two cell lines, may not be biologically relevant to acquired tamoxifen resistance.

When the minimum fold change value was set at two times the standard deviation of all protein ratios in the control sample, the total number of significantly changed proteins was reduced to 629 with 364 up-regulated and 265 down-regulated (Additional file 2 Table S2). Listed in Tables 1 and 2 are proteins selected for either their large fold changes or perceived relevance to breast cancer progression and adaptation to anti-estrogen treatment. Multiple, functionally distinct proteins are seen dramatically altered in their expression in the resistant cell line. Importantly, ER regulated proteins such as cathepsin D and trefoil factor1 (TFF1/PS2) were down-regulated, suggesting that suppression of ER signaling pathways is characteristic of tamoxifen resistance *in vitro*. Down-regulation of cathepsin D and TFF1/PS2 has also been reported in antihormone treated breast cancer cells [33].

Several of the up-regulated proteins are involved in the compensatory mechanisms for survival and proliferation in response to the anti-estrogen challenge. For example, up-regulation of TROP2 suggests increased survival signaling by activating ERK1/2 mediated cell cycle progression [34]. Overexpression of the antiapoptotic protein, CLU in the tamoxifen resistant cells suggests that it plays a role in counteracting the growth inhibition effects of tamoxifen.

Another group of differentially expressed proteins are associated with increased cancer cell motility and invasiveness, which include EphA2, BCAS1, S100 protein family members, Rho family members, Ral-A, Rab family members, Cdc42, MARCKS, Ezrin, Galectins 1 and 3 among others. These proteins are generally up-regulated and appear to regulate the cytoskeleton dynamics of the resistant cells leading to a more motile and aggressive phenotype.

To determine if the observed proteomic changes are due to acquired tamoxifen resistance or other changes including passaging the MCF-7 cells for 12 months, a three-way quantitative proteomic control experiment was performed in which an early passage #13 (week 12), mid passage #25 (week 27), and late passage #50 (week 58) MCF-7-control cells are compared. A total of 635 proteins were compared for their relative abundances by the fold-change ratios with statistical assessment (*P-*values) (Additional file 3 Table S3). These data confirm that there are no significant proteomic alterations within the MCF-7-control cells after a prolonged period of culture that are comparable to those occurring in the MCF-7-TamR cells. The relatively small fold changes in some protein expressions are not associated with the consistent, statistically significant changes occurring in the MCF-7-TamR resulting from development of resistance to tamoxifen. Overall, these data demonstrate that the progressive culturing of cells over a year in tamoxifen results in changes that are distinct from matched parental cells grown under normal culture medium conditions.

## Proteomic differential expressions are consistent with those at the transcriptional 2^nd ^el

**Figure 3:**
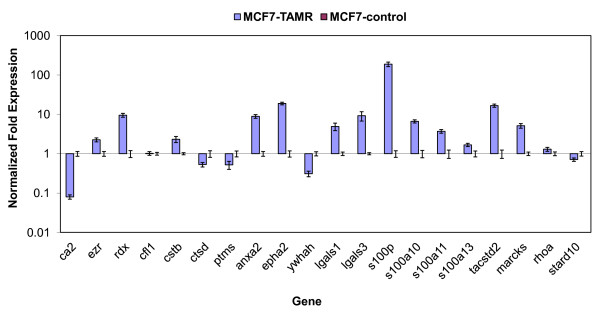
**Quantitative RT-PCR confirms that mRNA levels are consistent with protein expressions for the selected genes**.

To investigate whether the changes observed in protein expression are a result of transcriptional regulation, we performed quantitative real-time PCR of 20 differentially expressed proteins. Because expression levels of mRNA do not always parallel those of the proteins due to additional regulatory processes, such as post-transcriptional modifications, we sought to first validate the proteomic findings at the transcriptional level. As shown in Figure 3, changes in mRNA expression are consistent with proteomic fold changes. For example, the most prominently up-regulated gene, S100P (180-fold), was also one of the most significantly overexpressed proteins (5.2-fold). EphA2, a receptor tyrosine kinase that was overexpressed by nearly 3-fold in MCF-7-TamR, was up-regulated by 19-fold at the transcription level. Quantitative RT-PCR also confirmed the transcriptional down-regulation of several proteins whose concentrations were significantly decreased (for example, CA2, CTSD, starD10). Importantly, these results indicate that as a stable, tamoxifen resistant cell line, MCF-7-TamR has incurred extensive alterations in the proteome, and that these changes are paralleled at the transcriptional level.

### Western blots confirm proteomics fold changes

**Figure 4:**
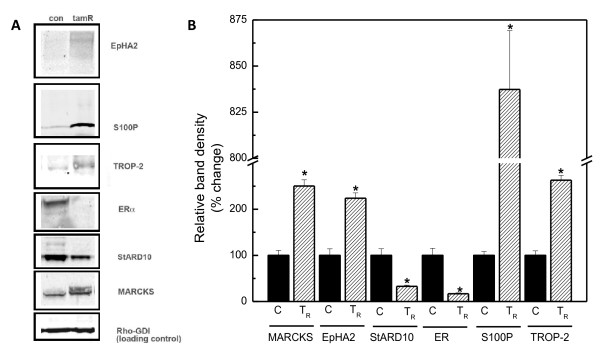
**Western blot analysis confirms protein changes in MCF-7-TamR cells initially identified in quantitative proteomics**. **A) **A representative Western blot of control (con) and tamoxifen resistant (MCF-7-TamR) cells showing the different expression levels of the indicated proteins. **B) **Four independent samples from each cell line were used to quantify the levels of each protein. The housekeeping protein Rho-GDI was used as an internal loading control. For comparative purposes, the control samples were set to 100%.

Recent advancement in proteomic techniques has made quantitative analysis of protein expression an ideal discovery tool with unprecedented reliability and breadth of scope. The multiple-channel labeling approach combined with high resolution mass spectrometry employed in this study provided an additional level of confidence and reproducibility to the proteomic results. However, when targets are narrowed down to individual functionally relevant proteins, Western blotting offers a more specific and efficient method of validation as long as antibodies are available. To this end, we sought to confirm our proteomic findings of some of the most significant targets by Western blot. Semi-quantitative Western blot analysis of the MCF-7-control and MCF-7-TamR cells was done for total protein levels of EphA2, S100P, TROP-2, StarD10 and MARCKS, all of which may be involved in the development of tamoxifen resistance. Results in Figure 4A, B show a statistically significant increase in the expression levels of EphA2 (approximately two-fold), S100P (approximately eight-fold), MARCKS (approximately three-fold), and TROP-2 (approximately three-fold) and a decrease in StarD10 (approximately two-fold) levels, confirming the differential expressions determined in both proteomic analysis and RT-PCR results.

Because proteomic analysis did not detect the presence and alterations of many receptors of key interests, including the tamoxifen target ER, we also performed Western blots to determine the status of ERα in the resistant cell line. Immunostaining clearly showed a marked decrease in total ERα protein level in the resistant cell line (> 20-fold), confirming that in the tamoxifen resistant MCF-7 cells obtained in our laboratory, ERα is significantly down-regulated but not lost (Figure 4A, B).

### ER regulated signaling pathways are suppressed but remain functional in MCF-7-TamR cells

**Figure 5:**
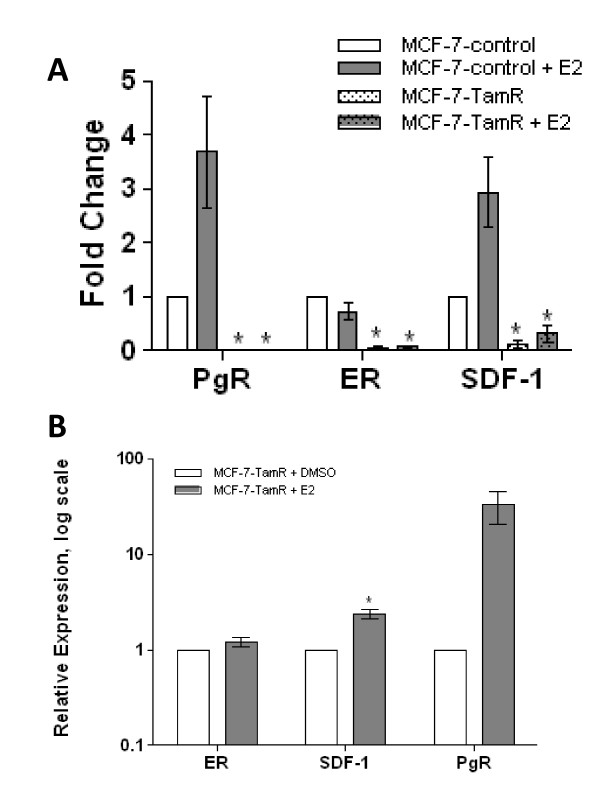
**MCF-7-TamR cells exhibit significantly down-regulated ER mediated signaling pathways**. **A) **MCF-7-control and MCF-7-TamR cells were grown in 5% phenol free DMEM for 48 hours prior to 24 hours treatment with 1 nM 17β-estradiol (E_2_) or vehicle control (DMSO). Q PCR was performed for ER regulated genes ER, PgR, and SDF-1. Normalization was to MCF-7-control cells treated with vehicle; **B) **E_2 _induced ER regulated gene expression for tamoxifen resistant cells (normalization to MCF-7-TAMR treated with DMSO). qPCR was performed for genes ER, SDF-1, and PgR. Results reflect average fold change in cycle number for mRNA levels +/-. Cycle number was normalized to β-actin. * Significantly different from DMSO control, *P *< 0.05.

The observation of reduced expression of ERα in the resistant MCF-7 cells prompted us to ask whether ER regulated signaling is suppressed and, if so, whether ER remains functional. We first investigated the expression of two ER regulated genes, PgR and SDF-1 in MCF-7-TamR and MCF-7-control cells (Figure 5A). Both PgR and SDF-1 were dramatically down-regulated in resistant cells when compared to DMSO treated control cells. For reference the ER gene expression was also determined by RT-PCR and was found to be greatly suppressed in the resistant cell line as its mRNA level was less than 10% that of the control. In MCF-7-control cells, treatment with E_2 _induced a three-fold increase in PgR mRNA level (Figure 5A). However, in the resistant cells, while the PgR level was low, E_2 _stimulation still caused a dramatic increase of PgR expression (Figure 5B). This observation indicated that ER remained functional in tamoxifen-resistant MCF-7 cells, albeit to a much diminished extent. Similarly, the mRNA expression of SDF-1, an ER dependent gene was seen significantly down-regulated in MCF-7-TamR cells compared to the control cells (Figure 5A). Upon treatment with E_2_, SDF-1 expression went up over two-fold, again suggesting that ER dependent signaling pathways remained functional after long term exposure to the anti-estrogen (Figure 5B).

### Pathway analysis reveals that actin cytoskeleton regulation drives enhanced cell motility in TamR cells

**Figure 6:**
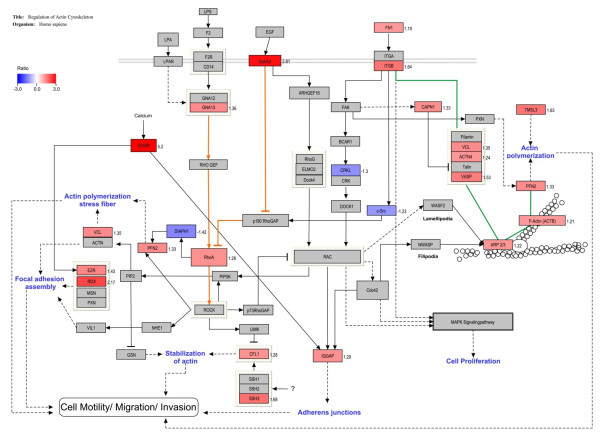
**Regulation of actin cytoskeleton in the tamoxifen resistant cell line, MCF-7-TamR**. The pathway illustrates changes to proteins expression, TamR cell line vs. control, involved in regulating the actin cytoskeleton. All red proteins are significantly up-regulated and all blue proteins are significantly down-regulated. The ratio value for each significantly regulated protein is indicated to the right. Solid lines with no arrow indicate physical interaction. Solid lines with an arrow or bar at the end indicate direct activation or inhibition, respectively. Dotted lines indicate indirect or unknown method of interaction. The orange lines highlight the Rho activation pathways and the green lines highlight integrin mediated focal adhesions.

Gene ontology analysis using PANTHER [25] indicates that the significantly changed proteins constitute an over representation of "Cytoskeletal regulation by Rho GTPase pathway" (*P *< 0.0001) and "Integrin signaling pathway" (*P *< 0.0001) (Additional file 4 Figure S4). To understand the molecular signaling associated with these proteomic changes in the tamoxifen resistant cells we mapped our protein changes on a custom pathway derived from an original KEGG pathway framework (See Experimental section). Twenty-four proteins from our proteomic data were identified as involved in the regulation of cell motility, of which 21 showed statistically significant changes in expression levels (ACTB, ARP2/3, c-Src, CAPN1, CFL1, CRKL, DIAPH1, EphA2, EZR FN1, GNA13, IQGAP, ITGB, PFN2, RhoA, RDX, S100P, SSH3, TMSL3, VASP, VCL). Figure 6 illustrates a reconstructed KEGG pathway map of actin cytoskeleton regulation. In one possible scenario, enhanced Rho-Rock signaling is enabled by increased expression of G-alpha 13 (GNA13) [35,36] and by EphA2-induced suppression of p190 RhoGap [37,38] (Figure 6, orange lines). In another signaling route depicted in the map, increased integrin-beta1 (ITGB) expression is implicated in the formation of focal adhesions with adaptor proteins talin, vinculin (VCL), actinin, filamin and other associated proteins such as vasodilator-stimulated phosphoprotein (VASP) [39]. This complex of integrins and proteins then binds to α-actin and f-actin through the Arp 2/3 complex. Up-regulation of several of these components indicate that the tamoxifen resistant cells are experiencing an increase of integrin mediated actin cytoskeleton regulation (Figure 6, green lines).

### MCF-7-TamR cells exhibit enhanced motility

**Figure 7:**
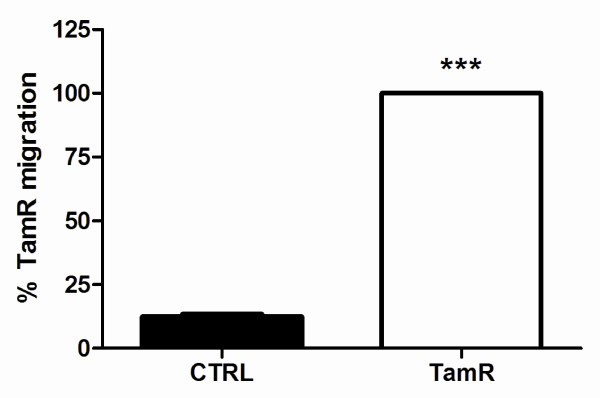
**Acquired resistance to tamoxifen in MCF-7 cells leads to enhanced migration capacity**. 2.5 × 10^4 ^MCF-7 or MCF-7-TamR cells were seeded on the upper chamber of a transwell system where the lower wells contained media with 5% serum. After 24 h, cells were fixed and stained with crystal violet and the number of migrated cells counted. Bars represent the percent of migrated MCF-TamR cells per 100 × field of view ± SEM. ***, *P *< 0.001.

The KEGG pathway analysis based on the proteomic data indicates that up-regulation of cytoskeleton related pathways may facilitate migration of MCF-7-TamR cells. To confirm this, we carried out transwell migration assays. When MCF-7-control and MCF-7-TamR cells were seeded at a density of 2.5 × 10^4 ^in media free of serum and phenol red, the tamoxifen resistant cells were found to migrate faster than the tamoxifen sensitive control cells. As shown in Figure 7, MCF-7-TamR cell demonstrated increased basal migration by eight-fold (100% migration compared to 13% for MCF-7-control cells). This result suggests that tamoxifen resistance is associated with enhanced cell motility, consistent with previous reports that antiestrogens promote breast cancer motility and invasion [12,40,41].

### S100P plays an important role in acquired tamoxifen resistance and enhanced cell motility

**Figure 8:**
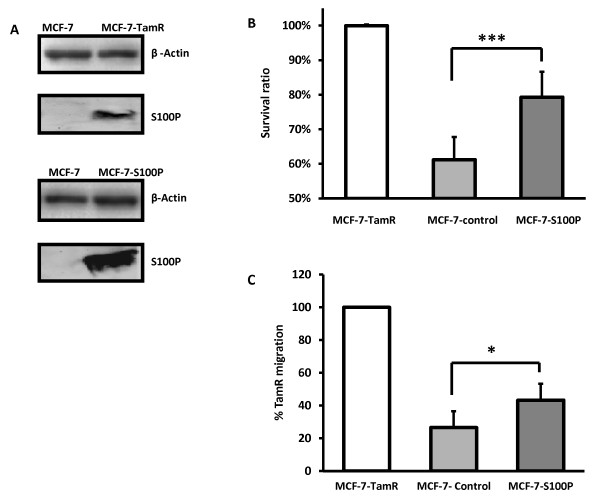
**S100P up-regulation in MCF-7 cells confers tamoxifen resistance and enhanced migration**. **A) **Western blot of S100P expression in MCF-7, MCF-7-TamR, MCF-7-S100P. **B) **Survival ratio of MCF-7, MCF-7-TamR, MCF-7-S100P after treatment of 4-hydroxytamoxifen for five days. **C) **Migration assay for MCF-7, MCF-7-TamR, MCF-7-S100P.

We next sought to investigate the role of S100P, a significantly up-regulated protein in MCF-7-TamR cells in conferring tamoxifen resistance and increased migration. As shown in Figure 8A, since the parental MCF-7 cell line expresses negligible level of S100P compared to the resistant cells, we decided to overexpress it in MCF-7 cells by a lentiviral transduction of the S100P gene. The resulting MCF-7-S100P cells exhibited a dramatic increase in S100P expression (Figure 8A). Subsequent survival assays demonstrated that stable overexpression of S100P in MCF-7 cells enhanced their resistance to tamoxifen when compared to the control. As illustrated in Figure 8B, after treatment with 4-OH Tam for five days at 10^-7 ^M, the survival ratio of MCF-7-S100P cells was significantly higher than the control MCF-7 cells (80% vs 60%, *P *< 0.001). The effect of S100P up-regulation on MCF-7 cell motility was also investigated by transwell migration assays. In Figure 8C, MCF-7 cells stably overexpressing S100P demonstrated over 60% (*P *< 0.05) increase in migratory capacity compared to the MCF-7-control cells.

### Survival analysis reveals relevance of many altered proteins to breast cancer prognosis

To assess the relevance of the altered expression levels of various proteins on the clinical outcome in breast cancer patients, we performed survival analysis of up- and down-regulated proteins selected in Tables 1 and 2 using an online survival analysis tool. The online database contains the expression of 22,277 genes and survival information of 1,809 patients [31]. As shown in the last columns of Tables 1 and 2, alterations in the expression level of many proteins in tamoxifen resistant cells were found to positively correlate with decreased survival. For example, the up-regulation of S100P, S100A10, S100A11, integrin alpha-V (itgav), macrophage-capping protein (capg), ezrin and RhoA appear to be predictive of poor survival (Table 1). On the other hand, down-regulation of a number of proteins such as proto-oncogene vav (vav1), trefoil factor 1 (tff1/PS2), translationally-controlled tumor protein (tpt1), glutathione S-transferase Mu 5 (gstm5), tyrosine-protein phosphatase non-receptor type 1 (ptpn1), and heat shock protein HSP 90-beta (hsp90ab1), are also significantly correlated to poor prognosis and decreased survival. However, tamoxifen resistance appears to induce expression changes of numerous proteins that are associated with improved survival in clinical results. For instance, the overexpression of breast carcinoma-amplified sequence 1 (bcas1), glutathione S-transferase Mu 1 (gstm1), ephrin A2 receptor (epha2), caveolin (cav), calpain small subunit 1 (capn2) and the down-regulation of stathmin (stmn1), serine-threonine kinase receptor-associated protein (strap), Ras-related protein Rap-1A (rap1a) all point to a better prognosis as indicated by the Kaplan-Meier survival curves (see negative long-rank *P-*values in the last columns of Tables 1 and 2).

**Figure 9:**
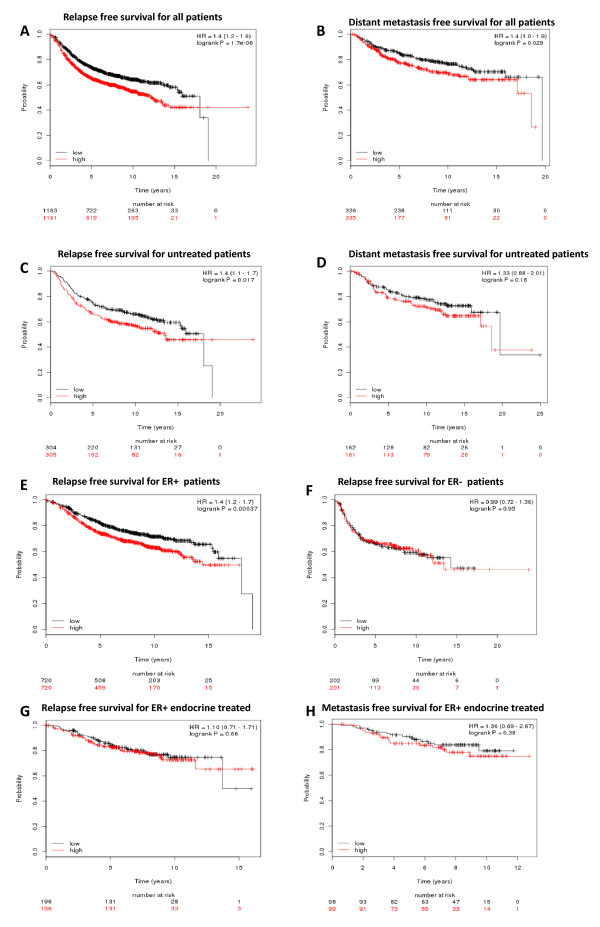
**Kaplan-Meier survival plots demonstrate the prognostic relevance of S100P overexpression on patient survival**. **A) **Overexpression of S100P is predictive of lower relapse free survival (*P *= 1.76e-6) for all patients; **B) **overexpression of S100P is correlated with decreased distant metastasis free survival (*P *= 0.029); **C) **Untreated breast cancer patients had lower relapse free survival if their tumors overexpressed S100P (*P *= 0.017); **D) **Weak correlation between the metastasis free survival and overexpression of S100P (*P *= 0.18); **E) **Higher level of S100P expression is predictive of poor relapse free survival for ER positive breast cancer patients (*P *= 0.00037); **F) **S100 overexpression is not associated with survival of ER negative breast cancer patients; **G) **S100 overexpression is not statistically significant for prognosis of ER positive endocrine treated patients; **H) **Overexpression of S100P is not predictive of metastasis-free survival of ER positive endocrine treated patients.

Figure 9A-D represents the Kaplan-Meier survival plots for S100P gene using two different survival options (Figure 9A, C) and two patient cohorts (Figure 9B, D). Up-regulation of S100P is correlated to reduced survival over a period of 20 years for both relapse free survival (*P *= 1.7e-6) and distant metastasis free survival (*P *= 0.029). For systematically untreated patients, overexpression of S100P gene is again predictive of lower relapse free survival rate (*P *= 0.017) but not statistically significant for prognosis of distant metastasis free survival (*P *= 0.18). Shown in Figure 9E, F are Kaplan-Meier survival curves where breast cancer subtyping is used based on ER status. For the ER (+) subgroup, overexpression of S100P is significantly associated with decreased survival (Figure 9E, P = 0.00037). However, this correlation is lost with ER(-) breast cancer patients (Figure 9F, P = 0.95), suggesting that S100P is not a useful predictor in hormone independent breast cancer subtypes. In addition, we found that the prognostic value of S100P in the available data set for ER+ endocrine treated patients (Figure 9G, H) was not significant.

## Discussion

We have established a tamoxifen-resistant breast cancer cell line obtained under an FBS-containing medium condition to minimize adaptive cellular changes in response to LTED. Indeed, earlier studies have shown that LTED leads to enhanced expression of the estrogen receptor [42] or EGFR [14], which are not usually observed in tamoxifen resistant cell lines cultured in normal FBS medium [13,15]. In the MCF-7-TamR cell line obtained in this study after six months of 4-OH tamoxifen treatment, the estrogen receptor was significantly down-regulated but retained viable function (Figure 5). Current understanding of endocrine resistance depicts a progressive, stepwise process in response to anti-estrogen challenge where breast cancer cells evolve from an estrogen-dependent phenotype to a non-responsive one and eventually to a stage of estrogen independence. Our results indicate the tamoxifen resistant cells appear to be at a stage of minimized estrogen responsiveness without complete loss of ER. Previous studies of tamoxifen resistance using *in vitro *models suggest translocation of ER from nucleus to membrane, facilitating crosstalk with growth factor receptors and enhancing the non-genomic signaling of the ER. In these reports, the total ER levels remain largely unchanged [13,15,42]. On the other hand, complete loss of ER expression has occurred when MCF-7 cells became resistant to the pure antiestrogen, fulvestrant [43-45].

This *in vitro *behavior is also consistent with clinical observations that tamoxifen resistant tumors may still respond to fulvestrant [46,47] and that only 15 to 30% of patients present with complete loss of ER at time of relapse [11,48,49]. The down-regulation of ER mediated signaling pathways in our MCF-7-TamR cells is corroborated by proteomic evidence that showed suppressed expression levels of cathepsin D and TFF1/PS2 and was confirmed by Western blot analysis showing diminished ER protein expression. PgR, an ER dependent gene, was also found significantly down-regulated (> 1,000-fold, Figure 5A) by RT-PCR analysis. On the other hand, E_2 _stimulation did induce a 50-fold increase in PgR expression from its greatly suppressed basal level (Figure 5B) in the resistant cells.

In ER positive breast cancer cells, estrogen signaling is the main mediator of proliferation and tumor progression. Adaptation to tamoxifen challenge which blocks ER signaling must involve activation of alternative survival signaling to sustain growth and circumvent the apoptotic effect of tamoxifen. As demonstrated in numerous *in vitro *and *in vivo *studies on the mechanisms of tamoxifen resistance, tumor cells recruit a remarkably wide variety of signaling pathways to achieve the resistant outcome [50,51], including cross talk with EGFR and Her2 [52,53], and enhanced nongenomic signaling accompanied by translocation of ER [54,55]. Our study identified several proteins that are known to promote tumorigenesis and progression but their roles in tamoxifen resistance have not been explored. In particular, the up-regulation of S100P revealed a previously unknown link between tamoxifen resistance and the small calcium binding protein. S100P is a ligand for the receptor for advanced glycation end product (RAGE). Binding of the Ca^2+ ^activated S100P homodimer to RAGE has been shown to promote cancer cell proliferation via the ERK1/2 and NFκB signaling pathways [56-58]. S100P was found to co-immunoprecipitate with RAGE and its action on cell survival and proliferation could be blocked by RAGE inhibitors [56].

The forced overexpression of S100P in the tamoxifen sensitive MCF-7 cell line increased its resistance to tamoxifen significantly (Figure 8B), confirming the role of S100P in acquired tamoxifen resistance. Our results suggest that, as the ER-regulated proliferation pathway was severely suppressed after prolonged exposure to tamoxifen, the S100P-RAGE signaling via activation of ERK1/2 and possibly NF-κB is increased as a compensatory mechanism of cell proliferation and survival.

In addition, the up-regulation of the anti-apoptotic protein CLU can be viewed as another possible survival pathway contributing to tamoxifen resistance. Previous reports have implicated CLU up-regulation as a general defense mechanism of cancer cells toward cytostatic drugs [59-61]. Under cell stress, such as treatment with trastuzamab in breast cancer cells, or following androgen ablation in prostate cancer cells, significant increase in CLU expression was associated with activation of alternative signaling [62,63].

Another significantly up-regulated protein, EphA2, may contribute to the survival of tamoxifen resistant cells. The EphA2 expression level in breast cancer cells has been found inversely related to ER expression [64,65]. This is consistent with our RT-PCR and Western blot results where ER was significantly down-regulated (Figures 4 and 5). EphA2-transfected cells demonstrated increased growth *in vitro *and form larger and more aggressive tumors *in vivo *[66]. Moreover, EphA2 overexpression decreased the ability of tamoxifen to inhibit breast cancer cell growth and tumorigenesis [67,68]. The finding in this study that EphA2 was overexpressed in a tamoxifen resistant cell line confirms the involvement of the receptor tyrosine kinase in the development of tamoxifen resistance in breast cancer.

As the cells adapt to the inhibitory effects of tamoxifen, the acquired resistance appears to transform the breast cancer cells into a more aggressive phenotype with increased motility. Indeed, many of the overexpressed proteins thought to regulate growth and proliferation in our TamR cells have also been implicated in promoting cancer cell migration and invasion. Gene Ontology and KEGG pathway analyses collectively using proteomic data suggest that regulation of actin cytoskeleton may be responsible for driving the motility of TamR cells. The novel role of S100P in the regulation of cytoskeleton dynamics was highlighted in the pathway map (Figure 6) in which S100P was involved in the interactions with ezrin [69], a membrane/F-actin cross-linking protein implicated in tumor metastasis [70-73], and with the scaffolding protein IQGAP1 [74], known to promote cell motility and invasion [75]. To confirm the involvement of S100P in regulation of tamoxifen induced cell motility, we conducted functional studies of S100P by overexpressing the protein in the parental MCF-7 cells and observed increased motility in MCF-7-S100P cells as a result (Figure 8C). Moreover, our proteomic finding that both ezrin and IQGAP1 were up-regulated in the tamoxifen resistant cells (1.43- and 1.29-fold, respectively) provided additional evidence for the involvement of S100P in motility enhancement and suggests that the mechanism of action may involve the ezrin and IQGAP1 pathways.

Finally, overexpression of S100P and its role in mediating tamoxifen resistance and cell motility also bear clinical relevance. Using a GEO gene expression database from 1,809 breast cancer patients, the Kaplan-Meier survival plots demonstrate the prognostic relevance of S100P overexpression on patient survival. Overexpression of S100P is predictive of lower relapse free survival (*P *= 1.76e-6) and significantly correlated with decreased distant metastasis free survival (*P *= 0.029). Furthermore, truly prognostic patient group, that is, systematically untreated breast cancer patients with higher levels of S100P tend to have shorter relapse free period (*P *= 0.017). Finally, S100P up-regulation appears to be significantly associated with reduced survival in ER(+) but not in ER(-) breast cancer patients.

## Conclusion

Using a quantitative proteomic approach we have identified and verified key adaptive protein changes that are involved in the development of tamoxifen resistance. Long term treatment with 4-hydroxytamoxifen significantly suppressed ER-regulated signaling pathways in MCF-7 breast cancer cells. This was demonstrated in the marked down-regulation of ER dependent genes, including PgR, PS2, and SDF-1. In response, alternative survival signaling was activated that appeared to involve the up-regulation of multiple proteins. This was reflected in the global proteomic changes that included the increased expression of TROP2, CLU, MARCKS, and S100 family proteins. In particular, we identified S100P, an EF-hand calcium binding protein previously implicated in breast and other solid tumors, as a significant player in conferring tamoxifen resistance and cell motility. Overexpression of S100P in the hormone sensitive parental MCF-7 cells significantly increased resistance to tamoxifen. The mechanism of S100P action may involve its interaction with the receptor RAGE, leading to sustained survival and proliferation.

Proteomic analysis of MCF-7-TamR cells also revealed a critical phenotypic transformation of the cells towards an increased migratory capacity, consistent with most clinical outcomes where tumor invasion and metastasis follow the acquired hormone resistance in patients. The enhanced cell motility in the tamoxifen resistant cells appeared to be driven by the cytoskeletal dynamics where S100P played an important role. This was supported by the observation that overexpressing S100P in MCF-7 cells significantly increased cell migration. Additional evidence comes from proteomic data where up-regulation of multiple proteins in a coordinated signaling network may regulate the actin cytoskeleton dynamics as depicted in our proposed pathway model. Specifically, we observed the up-regulation of EphA2, RhoA, ITGB1, vinculin, ezrin, and radixin, which are key proteins contributing to the increased cell motility in a tamoxifen resistant phenotype by promoting actin fiber polymerization, filopodia formation, and cell contractability.

## Abbreviations

actb: beta actin; anxa2: annexin 2; ca2: carbonic anhydrase 2; cfl1: cofilin; CID: collision-induced dissociation; CLU: clusterin; cstb: cystatin B; ctsd: cathepsin D; DCC: dextran coated charcoal-stripped; DMEM: Dulbecco's modified Eagle's medium; DMSO: dimethyl sulfoxide; EGFR: epidermal growth factor receptor; epha2: ephrin type-A receptor 2; ER: estrogen receptor; ezr: ezrin; HBSS: Hank's Buffered Salt Solution; HCD: high energy collision dissociation; lgals1: galectin-1; lgals3: galectin-3; ITGB: integrin-beta1; LTED: long term estrogen deprivation; marcks: myristoylated alanine-rich protein kinase C substrate; PgR: progesterone receptor; PMSF: phenylmethylsulfonyl fluoride; ptms: parathymosin; RAGE: receptor for advanced glycation end product; rdx: radixin; rhoa: Ras homolog gene family: member A; RFS: relapse free survival; Rho-GDI: Rho GDP-dissociation inhibitor; RT-PCR: reverse transcription polymerase chain reaction; s100a10: S100 protein A10; s100a11: S100 protein A11; s100a13: S100 protein A13; s100p: S100 protein P; SCX: strong cation exchange chromatography; SDF-1: stromal cell-derived factor-1; SPE: solid-phase extraction; stard10: StAR-related lipid transfer domain containing 10; Tam: tamoxifen; TCEP: tris(2-carboxyethyl)phosphine; TEAB: triethyl ammonium bicarbonate; TMT: tandem mass tag; ywhah: tacstd2 (trop-2): tumor-associated calium signal transducer 2; VASP: vasodilator-stimulated phosphoprotein; VCL: vinculin.

## Competing interests

The authors declare that they have no competing interests.

## Authors' contributions

CZ cultured cell lines, performed survival assays and proteomic sample preparation, RT-PCR, interpreted data and drafted the manuscript. QZ performed lentiviral transduction and subsequent functional studies of cell survival and migration, and contributed to drafting the revised manuscript. LR performed migration assays. IT performed bioinformatics analysis, while MRB performed Western blotting. QZ carried out HPLC-MS/MS based protein identification and database search. EM performed RT-PCR and SE performed colony assays. BMC and MEB participated in experimental design and interpretation, and critically revised the manuscript. GW designed the study, and drafted and critically revised the manuscript. All authors have read and approved the final manuscript.

## Supplementary Material

Additional file 1 **(Table S1). List of all proteins identified in global proteomic analysis**. The file contains 2,128 proteins that were identified and 2,088 proteins quantified, with IPI number, protein description, fold change and statistical *P-*value where applicable. Click here for file

Additional file 2 **(Table S2). List of significantly altered proteins from proteomic anlaysis**. This file contains 629 significantly changed proteins as defined in the Results section where the significant fold change of proteins was defined as greater than two times standard deviation and *P *< 0.05. Click here for file

Additional file 3 **(Table S3). List of proteins identified and quantified in three MCF-7-control cell samples obtained at different passages**. This file contains a total of 635 proteins with relative abundances by the fold-change ratios with statistical assessment (*P-*values). Click here for file

Additional file 4 **(Figure S4). Gene ontology analysis of significantly altered proteins reveals overrepresentation of Integrin Signaling Pathway**. This file contains the over or under representation of pathways determined using the web based program PANTHER http://www.pantherdb.org and the significantly up- and down-regulated proteins. Click here for file
