# Supplementary material for: Proteomic analysis of acquired tamoxifen resistance in MCF-7 cells reveals expression signatures associated with enhanced migration
Source: Breast Cancer Res. 2012 Mar 14;14(2):R45. doi: 10.1186/bcr3144 (PMC3446379; doi:10.1186/bcr3144)
Supplement: Additional file 4 — (Figure S4). Gene ontology analysis of significantly altered proteins reveals overrepresentation of Integrin Signaling Pathway. This file contains the over or under representation of pathways determined using the web based program PANTHER http://www.pantherdb.org and the significantly up- and down-regulated proteins. [file bcr3144-S4.DOC]

| Up Regulated Proteins |  |  |  |
| --- | --- | --- | --- |
| Pathway | Number of Proteins | Representation (Over/Under) | p-value |
| Cytoskeletal regulation by Rho GTPase | 11 | + | 1.65E-06 |
| Integrin signaling pathway | 13 | + | 2.29E-05 |
| Glycolysis | 5 | + | 8.78E-05 |
| Cortocotropin releasing factor receptor signaling pathway | 5 | + | 2.04E-04 |
| DNA replication | 4 | + | 5.45E-04 |
| Aminobutyrate degradation | 2 | + | 5.89E-04 |
| Huntington disease | 10 | + | 8.07E-04 |
| Beta3 adrenergic receptor signaling pathway | 4 | + | 1.20E-03 |
| Nicotinic acetylcholine receptor signaling pathway | 7 | + | 1.73E-03 |
| 5HT4 type receptor mediated signaling pathway | 4 | + | 2.26E-03 |
| TCA cycle | 3 | + | 3.42E-03 |
| Oxytocin receptor mediated signaling pathway | 5 | + | 4.28E-03 |
| Thyrotropin-releasing hormone receptor signaling pathway | 5 | + | 4.90E-03 |
| Gamma-aminobutyric acid synthesis | 2 | + | 5.06E-03 |
| 5HT2 type receptor mediated signaling pathway | 5 | + | 7.59E-03 |
| Beta2 adrenergic receptor signaling pathway | 4 | + | 7.70E-03 |
| Beta1 adrenergic receptor signaling pathway | 4 | + | 7.70E-03 |
| Pentose phosphate pathway | 2 | + | 8.79E-03 |
| Fructose galactose metabolism | 2 | + | 1.89E-02 |
| Muscarinic acetylcholine receptor 1 and 3 signaling pathway | 4 | + | 2.27E-02 |
| Pyruvate metabolism | 2 | + | 2.52E-02 |
| Inflammation mediated by chemokine and cytokine signaling pathway | 10 | + | 2.80E-02 |
| Pyrimidine Metabolism | 2 | + | 2.86E-02 |
| Heterotrimeric G-protein signaling pathway-Gq alpha and Go alpha mediated pathway | 6 | + | 3.10E-02 |
| Pyridoxal phosphate salvage pathway | 1 | + | 3.42E-02 |
| Leucine biosynthesis | 1 | + | 3.42E-02 |
| Alanine biosynthesis | 1 | + | 3.42E-02 |
| 5HT3 type receptor mediated signaling pathway | 2 | + | 3.98E-02 |
| 5-Hydroxytryptamine degredation | 2 | + | 4.80E-02 |
| Histamine H1 receptor mediated signaling pathway | 3 | + | 4.97E-02 |
|  |  |  |  |
| Down Regulated Proteins |  |  |  |
| Pathway | Number of Proteins | Representation (Over/Under) | p-value |
| Parkinson disease | 8 | + | 4.94E-05 |
| p53 pathway | 6 | + | 3.43E-03 |
| Cytoskeletal regulation by Rho GTPase | 5 | + | 8.65E-03 |
| O-antigen biosynthesis | 1 | + | 4.94E-02 |

Over or under representation of pathways was determined using the web based program PANTHER ([www.pantherdb.org](http://www.pantherdb.org/)). Lists of the significantly up and down regulated proteins was used an input. PANTHER applied gene ontology information to each protein and then used a binomial test to compare the number of proteins represented in a specific pathway to a reference list, generating a p-value. Only pathways with a p-value < 0.05 are shown.
